# High-fat diet-induced adipose tissue-resident macrophages, T cells, and dendritic cells modulate chronic inflammation and adipogenesis during obesity

**DOI:** 10.3389/fimmu.2025.1524544

**Published:** 2025-06-03

**Authors:** Mousumi Mandal, Md Abdullah Al Mamun, Ahmed Rakib, Udai P. Singh

**Affiliations:** Department of Pharmaceutical Sciences, College of Pharmacy, The University of Tennessee Health Science Center, Memphis, TN, United States

**Keywords:** obesity, inflammation, macrophages, T cells, dendritic cells, adipocytes, 3T3-L1

## Abstract

**Background:**

Obesity is one of the major healthcare challenges and socio-economic liabilities worldwide and is rapidly reaching pandemic proportions. Characterized by low-grade chronic inflammation in adipose tissue (AT), the development of obesity is influenced by genetic, neurologic, and metabolic factors, immune activation, and behavioral activities. During obesity, AT macrophages play a central role in inflammation, lipid metabolism, and mitochondrial function in adipocytes. In this study, we investigated how AT resident macrophages, T cells, and dendritic cells (DCs) communicate to coordinate and regulate AT inflammation during obesity.

**Methods:**

We performed contact mode *ex-vivo* co-culture of different combinations of AT resident immune cells from mice fed with high-fat diet (HFD) and normal diet (ND) and also 3T3-L1 adipocytes with macrophages, T cells, and DCs isolated from AT of mice fed HFD. We analyzed the expression of adiposity-associated genes, inflammatory markers, and levels of cytokines and chemokine in conditioned culture medium. We also analyzed adipogenesis and performed Oil Red O staining of co-cultured adipocytes to visualize lipid accumulation under these conditions.

**Results:**

We found that macrophages from AT derived from HFD-fed mice fueled adipogenesis and inflammation in 3T3-L1 adipocytes and stromal vascular fraction cells derived from ND AT. Macrophages from HFD AT also promoted the expression in ND-derived T cells of chemokines including CCL5 and CXCL10 and inflammatory cytokines including TNF-α, IL-1β, IFN-γ, and IL-17A. Interestingly, T cells from HFD AT also induced expression of inflammatory genes in ND macrophages and lipid accumulation and expression of inflammatory proteins like CXCL2, CCL3, and CCL4 in 3T3-L1 adipocytes. DCs also stimulated adipocyte differentiation, and expression of chemokines and inflammatory cytokines like CCL5, MCP-3, and TNF-α in 3T3-L1 adipocytes.

**Conclusions:**

Our findings suggest that during obesity, macrophages work together in a coordinated fashion to modulate the activities of T cells, stimulating adipocyte differentiation, and thereby sustaining chronic inflammation. Thus, macrophages in AT might serve as druggable targets in combatting obesity.

## Graphical Abstract

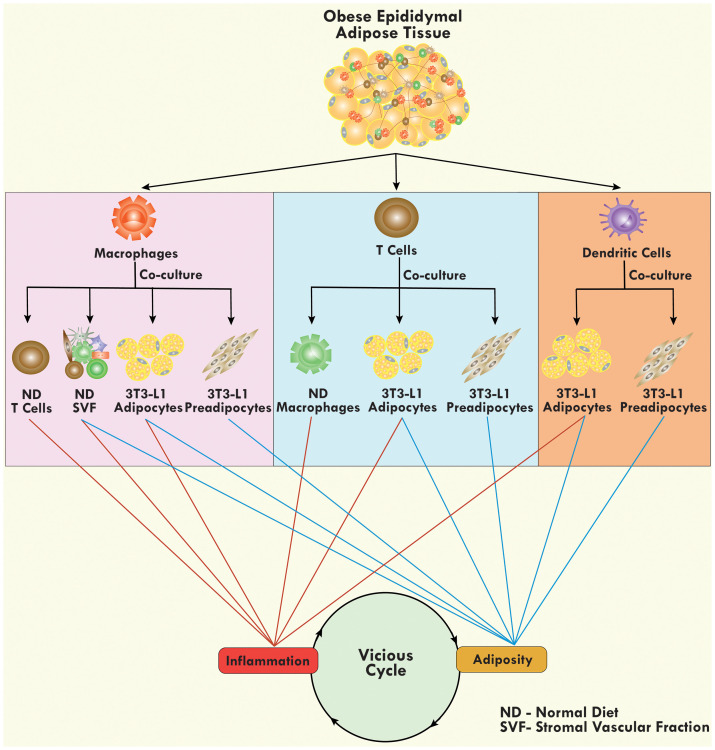

## Introduction

Obesity is one of the foremost healthcare burdens on the worldwide economy (1), representing the fifth most common cause of death globally and thus reaching pandemic proportions. Obese individuals are vulnerable to other diseases including atherosclerosis, hypertension, cardiovascular disease, fatty liver, metabolic disorders including type II diabetes mellitus (T2DM), and cancer (2, 3). Obesity is generally defined by elevated body mass index (BMI ≥ 30), atypical fat accumulation, and an aberrant surplus of energy (4) and is characterized by low-grade chronic inflammation, which paves the way for the development of several autoimmune diseases (5, 6). The imbalance between energy intake and expenditure results in a higher accumulation of triglyceride lipids in the adipose tissue (AT) and stimulates its expansion by hyperplasia and hypertrophy, leading to an increased number of adipocytes and increased adipocyte volume, respectively (7). Interestingly, obesity is more positively linked with hypertrophy, which induces the death of adipocytes (8) and also due to hypoxia and organelle stress (9). The death of adipocytes, AT inflammation, and dysfunction of metabolic processes are highly interlinked during obesity. Adipocyte death recruits immune cells to the AT, initially neutrophils and later macrophages that phagocytose the dead cells. In obesity, increased numbers of AT macrophages surround dead and dying adipocytes to form crown-like structures (CLS) (10). Macrophages in the CLS produce pro-inflammatory cytokines, including tumor necrosis factor-alpha (TNF-α), interleukin 1 beta (IL-1β), and IL-6, which attract more immune cells to the AT. T cells become predominant in AT (11) and are activated by potent antigen-presenting dendritic cells (DCs) (12), whose numbers also increase in obesity and chronic inflammation (13). Thus, during obesity, intricate crosstalk occurs among adipocytes, AT macrophages, T cells, and DCs, although there remains very limited information in the literature regarding cell-to-cell interactions in the AT and how these cells mediate inflammation, lipid accumulation, and adipogenesis in the AT milieu during obesity.

The most prominent means by which AT resident cells communicate with one another is via the secretion of cytokines, chemokines, and adipokines, which work together through their respective receptors on target cells. Both macrophages and adipocytes produce cytokines IL-1β, TNF-α, and IL-6, which regulate lipid metabolism, glucose transport, insulin resistance, and adipogenesis (14, 15). Other cytokines are also involved in this process. IL-12p70 produced by antigen-presenting cells is positively associated with obesity (16). Two other members of this family, IL-27 and IL-23 are primarily produced by DCs and activated macrophages (17). IL-23 mediates differentiation of T helper 17 (Th17) cells, which produce inflammatory cytokine IL-17A (18). IL-17A produced from Th17 cells and innate lymphoid cells promotes IL-6 production from adipocytes (19). Cytokine IFN-γ produced by T cells participates in AT inflammation by reducing the number of regulatory T cells in the AT (20). Chemokines including CXC chemokine ligand 1 (CXCL1), CXCL2, CXCL10, CC chemokine ligand 5 (CCL5), and CCL7 that are expressed in AT by immune cells, and adipocytes (21) serve to guide the migration of immune cells to the AT. Finally, chemokines like CCL5, monocyte chemoattractant protein-1 (MCP-1), and macrophage inflammatory protein-1 alpha (MIP-1α) are specifically secreted by adipocytes (22). Thus, it is crucial to explore how these cytokines and chemokines mediate the functions of macrophages, T cells, and DCs in the AT during HFD-induced obesity.

Several studies have reported the outcomes from transwell (non-contact) and contact mode co-culture systems in the context of obesity. To this end, it has been shown that human adipose resident lymphoid type-1 cells (ILC1s) boost adipose fibrosis and CD11c^+^ macrophage stimulation using *in vitro* transwell co-culture (23), and bone morphogenetic protein 7 of innate (ILC2) participate in differentiation of brown adipocyte when co-culture with mesenchymal stem cells (24). Further, activated T cells reduce pre-adipocyte to adipocyte differentiation in transwell co-culture (25), and indirect and direct co-cultures of macrophages with 3T3-L1 adipocytes attenuate insulin activity and change macrophage morphology and activation (26). Moreover, adipocytes trigger more AT resident CD4^+^ T cell activation via MHCII and leptin to fuel adipose inflammation in contact mode compared to the non-contact mode of co-culture (27). Thus, cytokine-chemokines are the regulatory factors for non-contact transwell-separated culture. On the other hand, secretory cytokine-chemokines and membrane-bound receptor-ligand interaction both are the guiding cues for outcomes of the contact mode of culture. In this study, we defined the crosstalk between immune cells, and adipocytes in the AT during obesity using a contact mode co-culture approach. We fed the mice a high-fat diet (HFD) for twelve weeks to generate a diet-induced obesity model, and control mice were fed a normal diet (ND). At the experimental endpoint, the mice were euthanized, and the AT stromal vascular fraction (SVF) was collected and used to isolate and purify macrophages, DCs, and T cells. AT resident macrophages from HFD-fed mice were co-cultured with T cells and SVF derived from ND-fed mice. Oppositely, AT resident T cells isolated from HFD-fed mice were co-cultured with macrophage from ND-fed mice. Further, we cultured AT resident macrophages, DCs, and T cells from HFD-fed mice with 3T3-L1 pre-adipocyte and adipocytes in various combinations to explore whether obese AT resident macrophages, T cells, and DCs induced adipogenesis by examining gene expression and lipid accumulation. We also estimated the levels of inflammatory cytokines and chemokines in co-culture media. Further, we examined whether HFD AT-derived macrophages, T cells, and DCs could induce the differentiation of pre-adipocytes to adipocytes and modulate adipogenesis.

## Materials and methods

### HFD-induced mouse model of obesity

Our use of the HFD-induced mouse model of obesity was approved by the University of Tennessee Health Science Center (UTHSC) Institutional Animal Care and Use Committee (IACUC) under protocol no. 23 – 0451. Wild-type (WT) C57BL/6 male mice (7–8 weeks of age) were procured from Jackson Laboratories (Bar Harbor, ME, USA) and housed under normal vivarium conditions with normal day/night cycles. The mice were kept at the animal facility for one week for acclimatization before initializing any dietary changes. Mice were in the chaw diet in the acclimatization phase (considered as 0 weeks). After one week of acclimatization, we measured the body weight and random blood glucose level before starting dietary intervention. We separated the mice into two experimental groups of five mice each (n=5/group) with comparatively higher body weight and blood glucose levels in the normal diet (ND) group and lower body weight and blood glucose levels in the high-fat diet (HFD) group to make more clarify that HFD is the only inducing factor for this obesity model. The ND group was fed a 10% kcal Research Diet (D12450J, New Brunswick, NJ) for twelve weeks, while the HFD group was fed 60% kcal) for twelve weeks. The ND contains protein (20% kcal), carbohydrate (70% kcal), fat (10% kcal), and in totality 3.85kcal/gm. On the contrary, HFD is composed of protein (20% kcal), carbohydrate (20% kcal), fat (60% kcal), and in totality 5.24 kcal/gm (Research New Brunswick, NJ). The feeding habits and body weight of each mouse were assessed weekly and their blood glucose levels were measured after eight weeks of feeding. However, we did not keep the mice in fasting condition or any other pre-conditions before measuring blood glucose levels. Hence, it is known as random blood glucose levels. Mice were euthanized after twelve weeks of diet feeding to collect the epididymal adipose tissue (eAT).

### Adipose tissue histology, hematoxylin, and eosin staining and imaging

A portion of the eAT was fixed in 4% paraformaldehyde for 24 hours at room temperature (RT), then dehydrated, embedded, and prepared the block in paraffin. eAT blocks were sectioned into 5 μm-thick slices, collected on glass slides, deparaffinized using xylene, and cleared using various concentrations of alcohol. Tissue sections were stained with hematoxylin and eosin (H&E) following standard protocols. The prepared, stained eAT sections were evaluated under a bright field microscope (Olympus, Japan) and images were captured.

### Stromal vascular fraction cells isolation from adipose tissue

The eAT from each mouse was collected with a mixture of DMEM (Cat. no. 10–027-CV, Corning, NY) and RPMI media (Cat. no. 10–041-CV, Corning, NY) supplemented with 10% fetal bovine serum (FBS) on ice. Tissues were minced in small pieces and incubated with shaking in 1mg/ml collagenase II (Cat. No. C6885, Sigma Aldrich, St. Louis, MO) at 37°C for 30 to 40 minutes. The same volume of ice-cold RPMI was added and the cell suspension was passed through a 100 μm filter on ice. The filtered cell suspension was centrifuged at 500 RCF, 8°C for 8 min. The cell pellet was incubated with ACK lysis buffer, resuspended in complete RPMI, and centrifuged again. The cell pellet was again resuspended in complete RPMI, and a single-cell suspension was prepared by passing through a 100 μm filter. Cells were counted in a cell counter (Invitrogen, USA) and kept on ice for further downstream experiments. We used 3–5 ND feed and 2–3 HFD feed mice AT to get sufficient numbers of stromal vascular fraction from ND and HFD groups respectively for downstream isolation of purified macrophages, T cells, and dendritic cells in MACS column separation technique (Miltenyi Biotec Auburn, CA).

### Isolation of T cells, macrophages, and dendritic cells from SVF

T cells, macrophages, and DCs were separated individually from SVF using a positive selection method using antibodies conjugated to magnetic microbeads (130-094-973, 130-049-601, and 130-125-835; Miltenyi Biotech), a MACS column (LD and LS) and a MACS separator (Miltenyi Biotech), according to the manufacturer’s protocol. Briefly, for each cell type, a portion of the SVF was incubated with the recommended ratio of microbeads and washed twice with the suggested buffer. The column was rinsed with buffer before applying the cell suspensions and placed in the MACS separator. Unbound cells were collected by washing the column with an appropriate amount of buffer. The column was removed from the MACS separator and cells of interest (> 96% pure) were collected in an appropriate amount of buffer. Further, it has been reported that Th1, Th17, and CD8^+^ T cells are the major T cell subsets that boost the AT inflammation (28). We isolated the total T cell using the magnetic microbeads separation technique by positive selection. Thus, it has been assumed that most of the inflammatory T cells are present in the HFD adipose tissue-resident T cell population.

### Co-culture of different combinations of immune cells and SVF

Macrophages and T cell populations were separately isolated from the SVF of mice fed ND or HFD as described above. T cells from HFD-fed mice (HT) with macrophages from ND-fed mice (NM) and macrophages from HFD-fed mice (HM) with T cells from ND-fed mice (NT) were seeded into six-well plates at the ratios of 0:1, 0.5:1, 1:1, and 2:1 and co-cultured in complete RPMI medium at 37°C, 5% CO_2_ for 72 h. Similarly, macrophages from HFD-fed mice (HM) were co-cultured with SVF from ND-fed mice (NSVF) at ratios of 0:1, 0.5:1, and 1:1. The HM, and HT are considered inducers, and the NM, NT, and NSVF are studied as target cells. Conditional medium (CM) was collected after 72 h from each co-culture and the cells were harvested for isolation of total RNA from every combination’s three biological repeats.

### Culture of 3T3-L1 pre-adipocytes and differentiation to adipocytes

The mouse pre-adipocyte cell line 3T3-L1 (ATCC-CL-173) was cultured, maintained, and expanded as described in our earlier study (29) and confluent 12 well plate culture was stimulated for 48 h with differentiation cocktail medium containing 0.5 mM isobutyl methylxanthine (IBMX), 1 mM dexamethasone, and 10µg/ml of insulin in DMEM + 10% FBS + 1% antibiotic solution to generate mature adipocytes. The cells were incubated with insulin (10µg/ml or 2.5µg/ml) in a complete DMEM medium for up to seven days. At this stage, most of the cells contained cytoplasmic oil droplets that were visible under a phase contrast microscope.

### Co-culture of immune cells with 3T3-L1 adipocytes and pre-adipocytes

T cells, macrophages, and dendritic cells (HDC) from HFD-fed mice were co-cultured with differentiated 3T3-L1 (D3T3-L1) adipocytes at the ratio of 0:1, 0.5:1, and 1:1 for 72 h. The HM, HT, and HDC are considered inducers, and D3T3-L1 adipocytes are studied as target cells. Conditioned media were collected and stored. Oil-red O staining was performed as described below, the cells were visualized and imaged under a bright field microscope, and total RNA was isolated from harvested cells. In parallel, T cells, macrophages, and dendritic cells from HFD-fed mice were also co-cultured with undifferentiated confluent pre-adipocytes (U3T3-L1) for six days either with (WD) or without (W/OD) the aforementioned differentiation cocktail medium. U3T3-L1 pre-adipocytes are used as target cells. The co-cultured 3T3-L1 cells morphology and the gradual deposition of oil droplets were regularly monitored under a phase contrast microscope and images of random fields in the 3T3-L1 cells co-culture plate were captured at the end of the experiment.

### Multiplex cytokine-chemokine assay of cultured medium

The co-culture media from each experiment were centrifuged at 1400 rpm for 10 minutes at 4°C and the supernatants were collected and stored frozen at -80°C until they were analyzed. We used a Mouse ProcartaPlex™ Mix & Match 19-plex (Cat. No. PPX-19-MXFVNWE, Thermo Fisher Scientific, USA) assay in a 96-well plate configuration to measure the concentrations of nineteen selected cytokines and chemokines in the conditioned media from different combinations of co-cultured cells, following the manufacturer’s protocol. In brief, CM was thawed, standards were prepared in medium following the recommended protocol, and the recommended volume of capture beads was added to each. Standard and CM were added in pre-defined wells; the blank well contained only medium. The 96-well plate was incubated with shaking overnight at 4°C. The next day, the plate was washed three times and incubated with detection antibodies for 1 h at RT. Later, the plate was incubated with streptavidin-phycoerythrin (PE) at RT for 30 min. The beads were then resuspended in the suggested buffer and analyzed immediately using a Luminex™ System (Austin, TX) with analytic software from Bio-Rad (Hercules, CA). The results were stated as picograms per milliliter (pg/ml).

### RNA isolation and RT-qPCR analysis

The cells were washed twice with ice-cold phosphate buffer saline (PBS), harvested, and centrifuged to pellet the cells. Total RNA was extracted from cells using the RNeasy Mini kit (QIAGEN, Germantown, MD) following the manufacturer’s protocol and described in detail in our previous study (30). A Nanodrop spectrophotometer (Fisher Scientific) was used to determine the absorbance at wavelengths of 260 and 280 nm and the concentration and relative purity (A260/A280 ratio) of the RNAs were calculated. For each sample, 250 ng RNA was used as a template for first-strand cDNA synthesis by reverse transcriptase, the complementary strand was synthesized with DNA polymerase using an iScript cDNA synthesis kit, and the cDNA was analyzed using quantitative PCR (qPCR), as described in our earlier study (30). The details of the primers we used are provided in **Table 1**; all primers were purchased from Integrated DNA Technologies (IDT; Coralville, IA, USA).

**Table 1:** List of primer sequences for RT-qPCR.

Name of the Gene	Sequence (5′ to 3′)	
*Cd11b*	For	TACTTCGGGCAGTCTCTGAGTG
	Rev	ATGGTTGCCTCCAGTCTCAGCA
*F4/80*	For	CGTGTTGTTGGTGGCACTGTGA
	Rev	CCACATCAGTGTTCCAGGAGAC
*Gapdh*	For	GAAGCCCATCACCATCTT
	Rev	CAGTAGACTCCACGACATAC
*Il6*	For	CAGAGTCCTTCAGAGAGATAC
	Rev	ATGGTCTTGGTCCTTAGC
*MCP1*	For	GAATGGGTCCAGACATACA
	Rev	CAGATTTACGGGTCAACTTC
*Cd206*	For	GTTCACCTGGAGTGATGGTTCTC
	Rev	AGGACATGCCAGGGTCACCTTT
*Il1b*	For	TGGACCTTCCAGGATGAG GACA
	Rev	GTTCATCTCGGAGCCTGTAGTG
*Arginase1*	For	CATTGGCTTGCGAGACGTAGAC
	Rev	GCTGAAGGTCTCTTCCATCACC
*Nfkb*	For	GAAGACACGAGGCTACAA
	Rev	GGAAGGCATTGTTCAGTATC
*Stat3*	For	AGGAGTCTAACAACGGCAGCCT
	Rev	GTGGTACACCTCAGTCTCGAAG
*GATA3*	For	CCTCTGGAGGAGGAACGCTAAT
	Rev	GTTTCGGGTCTGGATGCCTTCT
*Ifng*	For	GACCTAGAGAAGACACATCAG
	Rev	AACAGCCATGAGGAAGAG
*Tnfa*	For	GGTGCCTATGTCTCAGCCTCTT
	Rev	GCCATAGAACTGATGAGAGGGAG
*Leptin*	For	GCAGTGCCTATCCAGAAAGTCC
	Rev	GGAATGAAGTCCAAGCCAGTGAC
*Pparg*	For	CCAAAGTGCGATCAAAGTAG
	Rev	CCATGAGGGAGTTAGAAGG
*Cebpa*	For	CAGGAGGAAGATACAGGAAG
	Rev	AGGACACAGACTCAAATCC
*Fasn*	For	CACAGTGCTCAAAGGACATGCC
	Rev	CACCAGGTGTAGTGCCTTCCTC
*Glut4*	For	CTTGGCTTCTTCATCTTCAC
	Rev	GTTTCACCTCCTGCTCTAA
*Pgc1a*	For	GCGGACAGAATTGAGAGA
	Rev	ACGGTAGGTGATGAAACC
*Resistin*	For	GGGAATTGTGTGGGAAATG
	Rev	GAGAGTCTCAAAGAGGAAGG

Primers were purchased from IDT, Coralville, IA, USA.

Cd, Cluster of differentiation; Gapdh, Glyceraldehyde-3-phosphate dehydrogenase; Il, interleukin; MCP1, Monocyte chemoattractant protein-1; Nfkb, Nuclear Factor kappa-light-chain-enhancer of activated B cells; Stat3, Signal transducer and activator of transcription 3; GATA3, GATA binding protein 3; Ifng, Interferon gamma; Tnfa, Tumor necrosis factor; Pparg, Peroxisome proliferator activated receptor gamma; Cebpa, CCAAT enhancer binding protein alpha; Fasn, Fatty acid synthase; Glut4, Glucose Transporter 4; Pgc1a, Peroxisome proliferator-activated receptor gamma coactivator 1 alpha.

### Oil Red O staining and imaging

Oil Red O (ORO) staining was performed to visualize oil droplets in 3T3-L1 adipocytes, as described previously (31). Briefly, 3T3-L1 adipocytes co-cultured with different immune cells isolated from HFD-fed mice were fixed with 4% paraformaldehyde, stained with freshly prepared ORO followed by staining with hematoxylin, and the excess stain was removed by washing with distilled water. The cells were kept in PBS until they were visualized and imaged under a bright-field microscope.

### Statistical analysis

Data are displayed as mean values ± standard error of the mean (SEM) from three experimental replicates. Statistical analysis was performed using an unpaired t-test and one-way analysis of variance (ANOVA) followed by Dunnett’s multiple comparison test where applicable to verify the significance level and p-values of 0.05 and below were taken as statistically significant in all analyses. The p-values were reported in the figures as *p < 0.05, **p < 0.01, ***p < 0.001, and ****p < 0.0001. All graphical plots were created using GraphPad Prism software (GraphPad Software, Boston, MA).

## Results

### Feeding mice on a high-fat diet increased body weight, blood glucose, adipocyte size, and the frequency of crown-like structures in adipocytes

**Figure 1:**
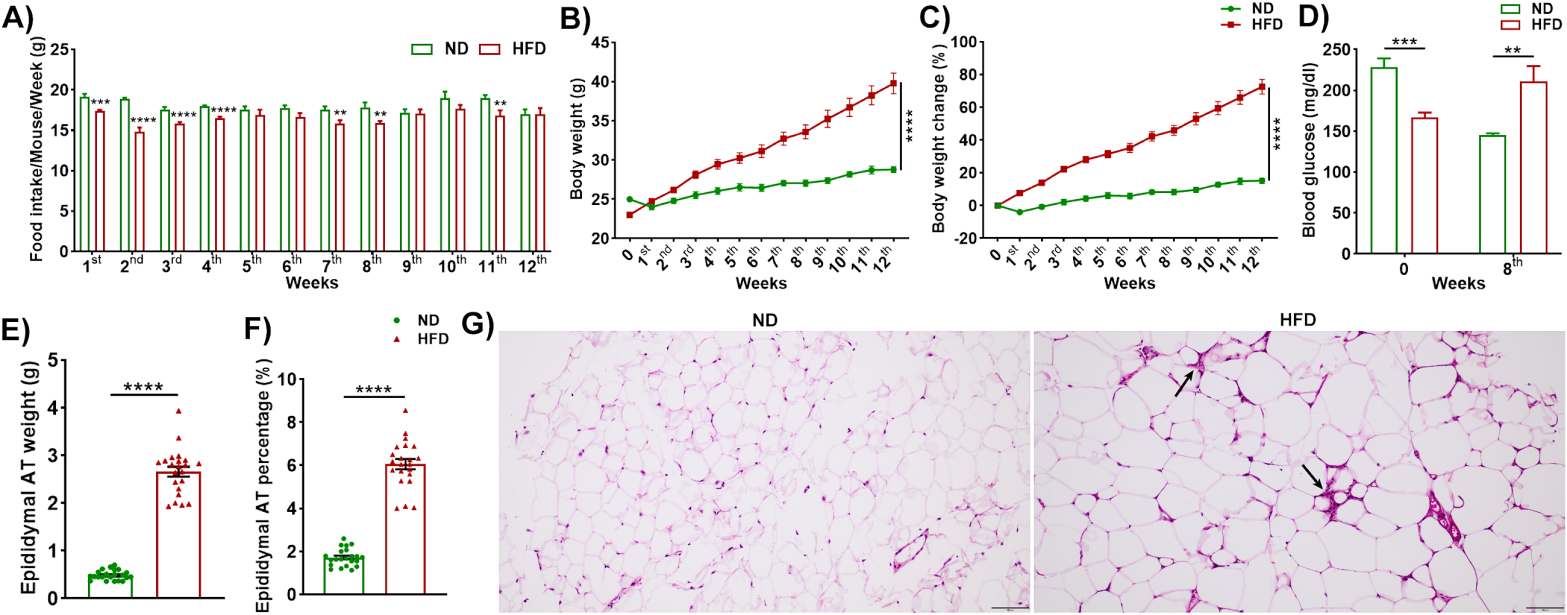
Feeding mice on a high-fat diet (HFD) increased body weight, blood glucose levels, epididymal adipose tissue weight, and adipocyte size, and developed more crown-like structures (CLS). Wildtype (WT) mice were fed a normal diet (ND) or high-fat diet for twelve weeks. **(A)** Average food consumption of a mouse per week. **(B)** Body weight from weeks 0 to 12. **(C)** Percent (%) body weight change from weeks 0 to 12. **(D)** Blood glucose levels before (week 0) and after 8 weeks of feeding ND and HFD. **(E)** Epididymal adipose tissue (eAT) weight. **(F)** Percentage of eAT weight relative to body weight. **(G)** Alteration of adipocyte size and numbers of CLS (marked by black arrow) in epididymal adipocytes visible on histological sections stained with hematoxylin and eosin (H&E); scale bar, 100 μm. **(A-C)**, n = 15; **(D)**, n = 5; **(E, F)**, n = 22. Data are presented as mean values ± SEM; statistical analyses were performed by unpaired t-test: **p < 0.01, ***p < 0.001, and ****p < 0.0001.

We fed wild-type mice on either a high-fat diet (HFD; 60% kcal from fat) or a normal diet (ND; 10% kcal from fat) for twelve weeks and measured food intake and body weight weekly. Interestingly, mice in the HFD group consumed a lesser amount (weight) of food compared to those in the ND group (**Figure 1A**). However, the HFD-fed mice gained more weight and exhibited a greater percentage weight change than mice in the group fed an ND (**Figures 1B, C**). During week 0, the mice in the HFD group exhibited lower blood glucose levels than those in the ND group, but significantly increased during eight weeks of HFD feeding (**Figure 1D**). The observed difference in glucose level in 0 week might be due to the relatively higher body weight in ND mice before dietary intervention. The body weight scenario depicted that ND’s initial body (25 ± 0.14 g) was higher than HFD (23 ± 0.24 g) in (**Figure 1B**). Mice are nocturnal and mostly feed at night. However, we measured the random blood glucose in the daytime. The study showed a prominent fluctuation in blood glucose between an hour of interval random and fasting in a low-fat diet and a high-fat diet (32). Fasting glucose levels might focus better on this issue. The weight of the epididymal AT was markedly increased by nearly five-fold in HFD-fed mice relative to ND-fed mice (**Figure 1E**). The eAT contributed to 6.05 ± 1.11% of body weight in the mice fed HFD, while it contributed 1.72 ± 0.09% of body weight in the ND-fed mice (**Figure 1F**). We observed that adipocyte size of HFD eAT increased compared to ND eAT in light microscopic images of H&E-stained AT sections (**Figure 1G**). The HFD-fed mice had larger adipocytes that contained a greater number of crown-like structures compared to those of the mice fed ND, which suggests that there was a higher level of adipocyte death and more infiltration of immune cells in the eAT of the HFD-fed group (**Figure 1G**). Together, these results indicate that feeding mice with HFD elevated body weight, increased glucose levels adipocyte size, and infiltration of the AT by immune cells consistent with diet-induced obesity.

### T cells from HFD-fed mice altered the gene expression and inflammatory response in macrophages from ND-fed mice

**Figure 2:**
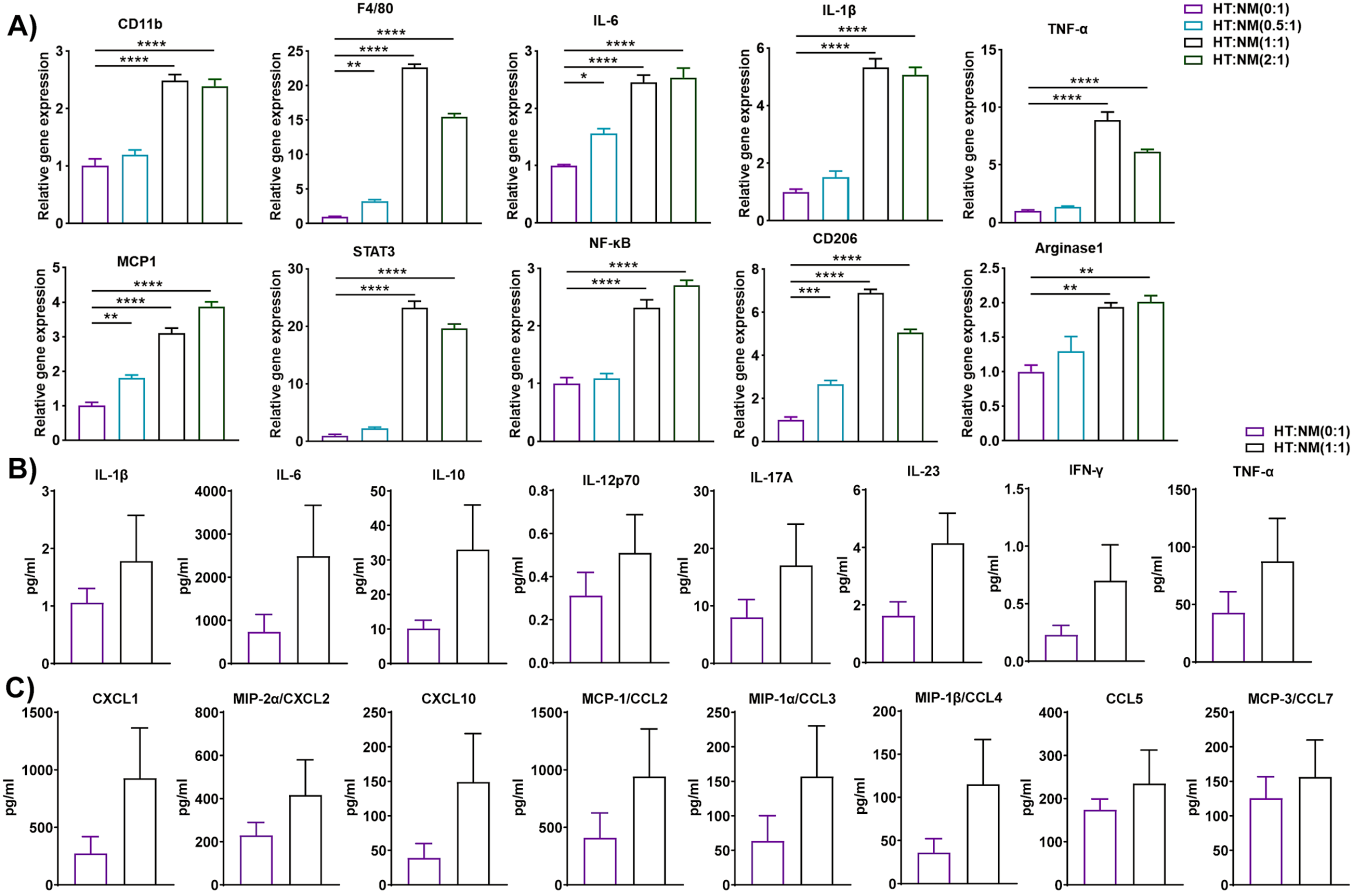
T cells from HFD-fed mice regulated gene and protein expression in macrophages from ND-fed mice. Mice were fed ND or HFD for twelve weeks. T cells and macrophages were isolated from the epididymal AT SVF of HFD and ND mice, respectively. HFD T cells and ND macrophages were co-cultured at a ratio of 0:1, 0.5:1, 1:1, and 2:1 for 72 h, with cells cultured at a 0:1 ratio serving as a macrophage control. Cell culture conditioned medium (CM) was collected and stored for later measurement of cytokine and chemokine levels, cells were harvested, and total RNA was isolated for **(A)** analysis of macrophage-specific genes and inflammatory markers by RT-qPCR. **(B, C)** For cells co-cultured at a 0:1 and 1:1 ratio, levels of **(B)** cytokines and **(C)** chemokines in the culture medium were measured. Data are presented as mean values ± SEM; statistical analysis was performed using **(A)** one-way ANOVA then Dunnett’s *post hoc* test or **(B, C)** unpaired t-test (n = 3), *p < 0.05, **p < 0.01, ***p < 0.001, ****p < 0.0001. HT, AT-derived T-cells from HFD-fed mice; NM, AT-derived macrophages from ND-fed mice.

T cell-derived IFN-γ is a crucial player in the conversion of the macrophage phenotype from anti-inflammatory to pro-inflammatory (33). Thus, we isolated and purified T cells from the eAT of HFD-fed mice and macrophages from ND-fed mice and co-cultured them at a T cell: macrophage ratio of 0:1, 0.5:1, 1:1, and 2:1 for 72 h. HT and NM are considered inducer and target cells respectively in this set of co-cultures. When we isolated total RNA from these cells and performed RT-qPCR analysis, we found that the expression of CD11b, F4/80, CD206, and arginase1 was increased in co-cultured cells related to control cells (**Figure 2A**). The expression levels of inflammatory markers IL-6, IL-1β, TNF-α, MCP-1, STAT3, and NF-κB were also higher in co-cultured cells, particularly at a ratio of 1:1 and 2:1 (**Figure 2A**). Besides, 1:1 ratio showed more elevated expression of several markers like F4/80, TNF-α, STAT3, and CD206 compared to 2:1 ratio. It is well known that AT resident regulatory T (Treg) cell population decreases during obesity compared to other T cell subsets (34). In our experiment, we used a total adipose tissue-resident T cell population from HFD mice. Technically, there was a greater number of total T cells when we cultured HFD T cells: ND macrophage in a 2:1 ratio compared to a 1:1 group. It also increased the chance of the presence of more Treg cells in the population in a 2:1 ratio compared to a 1:1 ratio. The increment of Tregs might suppress macrophage activation and result in decreased levels of F4/80, TNF-α, and STAT3. The levels of cytokines and chemokines in a conditioned co-culture medium also increased, although the difference was not statistically significant when the cells were co-cultured at a 1:1 ratio relative to controls (**Figures 2B, C**). In this study gene expression levels show a difference, but cytokine levels in media are not significantly altered. The inconsistencies between mRNA expression and the related secreted protein concentrations of chemokines indicate that mRNA expression may not always true indicator of protein loads. Remarkably, while the study has reported a correlation between the RNA transcript and protein concentration of certain chemokines (35), others have noticed that post-transcriptional modification can meaningfully impact protein secretion exclusive of disturbing mRNA expression (36). The co-culture approach is a dynamic system where two different cell types interact with each other compared to monoculture. This indicates that mRNA levels may not always forecast protein expression, due to the presence of various regulatory processes of post-transcription. Taken, together, the results indicate that HFD AT resident T cells participate in the alteration of the macrophage inflammatory phenotype and function during diet-induced obesity.

### Macrophages derived from HFD-fed mice modulated the inflammatory gene of ND T cells

**Figure 3:**
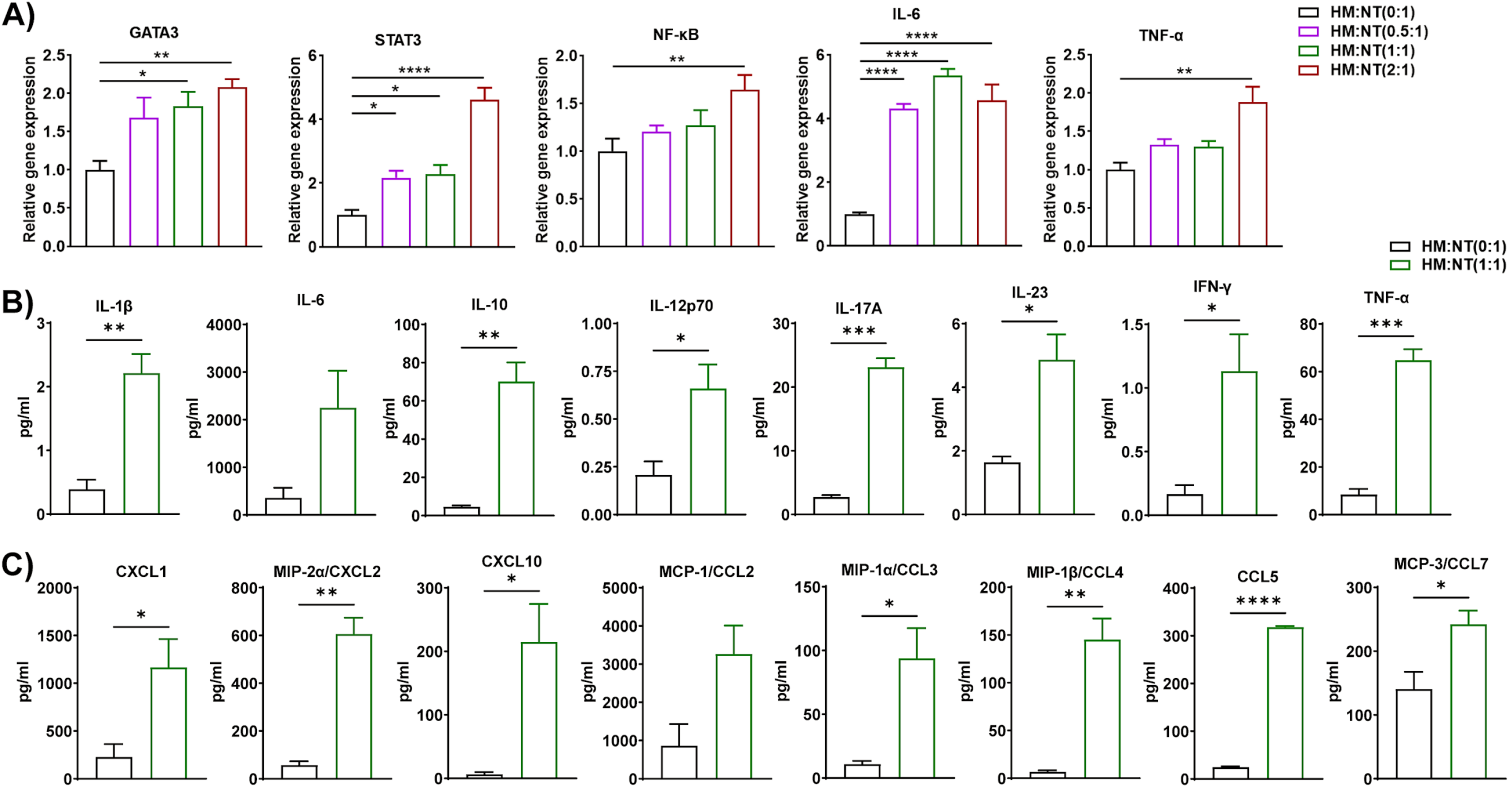
Macrophages from HFD-fed mice stimulated the expression of inflammatory genes and proteins in T cells from ND-fed mice. Mice were fed ND or HFD for twelve weeks. Macrophages and T cells were isolated from the epididymal AT SVF of HFD and ND mice, respectively. Macrophages from HFD-fed mice and T cells from ND-fed mice were co-cultured for 72 h at a ratio of 0:1, 0.5:1, 1:1, and 2:1 for 72 h, with cells cultured at a 0:1 ratio serving as a T cell control. CM was collected and stored for later measurement of cytokine and chemokine levels, cells were harvested, and total RNA was isolated for **(A)** analysis of T cell-specific genes and inflammatory markers by RT-qPCR. **(B, C)** For cells co-cultured at a 0:1 and 1:1 ratio, levels of **(B)** cytokines and **(C)** chemokines in CM were measured. Data are presented as mean values ± SEM; statistical analysis was performed using **(A)** one-way ANOVA then Dunnett’s *post hoc* test or **(B, C)** unpaired t-test (n = 3), *p < 0.05, **p < 0.01, ***p < 0.001, ****p < 0.0001. HM, AT-derived macrophages from HFD-fed mice; NT, AT-derived T cells from ND-fed mice.

Macrophages are critical regulators of low-grade chronic inflammation associated with obesity (37). Thus, we isolated macrophages from the eAT of HFD-fed mice and T cells from ND-fed mice and co-cultured them for 72 h at varying macrophage to T cell ratios (0:1, 0.5:1, 1:1, and 2:1). HM and NT is considered as inducer and target cells respectively in this combination of co-culture. As before, conditioned media were collected from each co-culture and stored, while the cells were harvested and total RNA was isolated and analyzed by RT-qPCR. We found that expression of the T cell differentiation marker GATA binding protein 3 (GATA3) and the inflammatory markers STAT3, NF-κB, IL-6, and TNF-α increased when these cells were co-cultured, primarily when they were combined at ratios of 1:1 and 2:1 (**Figure 3A**). The levels of IL-1β, IL-10, IL-12p70, IL-17A, IL-23, IFN-γ, and TNF-α in conditioned medium collected from these co-cultures increased relative to controls when the cells were cultured at a ratio 1:1 (**Figure 3B**). Interestingly, levels of the chemokines CXCL1, CXCL2, CXCL10, CCL3, CCL4, CCL5, and CCL7 were also higher in CM of macrophages from HFD-fed mice co-cultured at a ratio of 1:1 with T cells from ND-fed mice (**Figure 3C**). These results suggest that macrophages derived from the eAT of HFD-fed mice stimulate the expression of inflammation and differentiation markers in co-cultured T cells, which might be key to maintaining AT inflammation during obesity.

### Macrophages from HFD-fed mice elicited gene and protein expression in stromal vascular fraction cells from ND-fed mice

**Figure 4:**
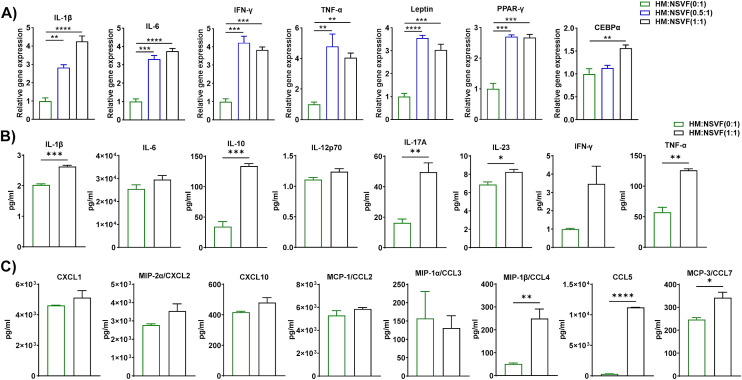
Macrophages from HFD-fed mice induced gene and protein expression in cells from the stromal vascular fraction of ND-fed mice. Mice were fed ND or HFD for twelve weeks. Macrophages were isolated from the epididymal AT SVF of HFD-fed mice and co-cultured for 72 h with cells of the SVF of ND-fed mice at a ratio of 0:1, 0.5:1, and 1:1, with cells cultured at a 0:1 ratio serving as a control. CM was collected and stored for later measurement of cytokine and chemokine levels, cells were harvested and total RNA was isolated for **(A)** analysis of inflammatory and adipogenic markers by RT-qPCR. **(B, C)** For cells co-cultured at a 0:1 and 1:1 ratio, levels of **(B)** cytokines and **(C)** chemokines in CM were measured. Data are presented as mean values ± SEM; statistical analysis was performed using **(A)** one-way ANOVA then Dunnett’s *post hoc* test or **(B, C)** unpaired t-test (n = 3), *p < 0.05, **p < 0.01, ***p < 0.001, and ****p < 0.0001. HM, AT-derived macrophages from HFD-fed mice; NSVF, AT-derived SVF from ND-fed mice.

AT is composed of non-adipocytes, namely fibroblasts, mesenchymal stem cells, endothelial cells, pericytes, and immune cells, which are collectively known as the stromal vascular fraction (38). Indeed, several members of SVF are the progenitor cells of adipocytes. We proposed to explore whether HFD macrophages play a role in adipocyte precursor to adipocyte differentiation. In this co-culture set, macrophages were inducer and SVF cells were considered as targets. Therefore, we evaluated the adipocyte differentiation-associated genes like PPAR-γ, and CEBPα and adipokines like leptin, TNF-α, IL-6, etc. Leptin is an adipokine regulator of obesity acts as a metabolic regulator and shows a biphasic role in pre-adipocyte proliferation (39). The proliferation of porcine pre-adipocytes was elevated during higher levels of external leptin (40), while a lower dose of leptin was required to induce rat preadipocyte cell proliferation (41). It has been shown that PPARγ is a nuclear hormone receptor and a central regulator transcription factor for adipocyte differentiation (42). Further, C/EBPα and PPARγ contribute to the same pathway for the generation of mature adipocytes (43). Additionally, eAT is a vital source of IL-6 which executes the pleiotropic function. Pre-adipocytes and immune cells especially macrophages in SVF produce IL-6 which is responsible for obese AT inflammation (44). We co-cultured macrophages from HFD-fed mice for 72 h with SVF cells of ND-fed mice at a macrophage to SVF ratio of 0:1, 0.5:1, and 1:1. HM and NSVF are considered as inducer and target cells respectively in this blend of co-culture. As before, we isolated total RNA for RT-qPCR analysis, and CM samples were stored to measure levels of cytokines and chemokines. Subsequent RT-qPCR analysis revealed increased expression of inflammatory genes IL-1β, IL-6, IFN-γ, TNF-α, and leptin, primarily when the cells were co-cultured at ratios of 0.5:1 and 1:1, and that expression of the adipocyte differentiation markers PPAR-γ and CEBPα were upregulated when the cells were co-cultured at a 1:1 ratio and PPAR-γ at 0.5:1 ratio also (**Figure 4A**). We also measured the levels of cytokines and chemokines in the CM when macrophages from HFD-fed mice were co-cultured with SVF cells from ND-fed mice at ratios of 0:1 and 1:1. The levels of IL-1β, IL-10, IL-17A, IL-23, and TNF-α increased when these cells were plated at a 1:1 ratio, relative to those detected at a 0:1 ratio (**Figure 4B**). Among the nine chemokines assayed, only CCL4, CCL5, and CCL7 were upregulated in CM from a 1:1 co-culture (**Figure 4C**). Our results suggest that eAT-resident macrophages from HFD-fed mice induced SVF immune cells from ND-fed mice to become pro-inflammatory and stimulated differentiation of pre-adipocytes to adipocytes during HFD-induced obesity and consistent with other previous studies.

### Macrophages isolated from HFD-fed mice accelerated lipid accumulation, adipogenesis, and inflammation in 3T3-L1 adipocytes

**Figure 5:**
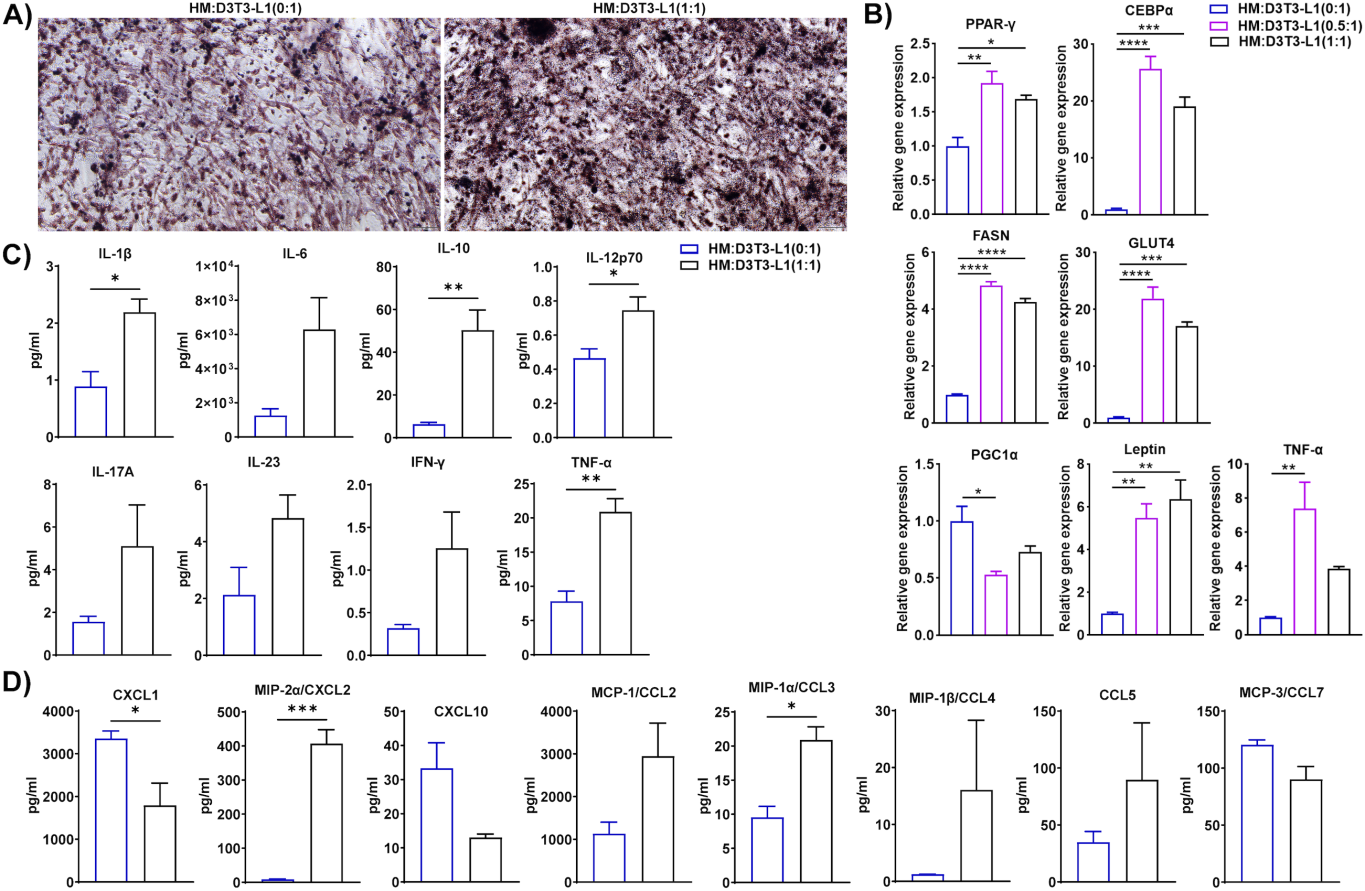
Macrophages from HFD-fed mice induced lipid accumulation, adipogenesis, and inflammation in 3T3-L1 adipocytes. Mice were fed HFD for twelve weeks. Macrophages were isolated from SVF of epididymal AT of HFD. 3T3-L1 pre-adipocytes were cultured and differentiated into adipocytes. HFD macrophage and 3T3-L1 adipocytes were co-cultured in the ratio of (0:1, 0.5:1, and 1:1) for 72 h, and 0:1 ratio was considered as control. CM was collected and stored for later measurement of cytokine and chemokine levels and **(A)** the cells co-cultured at a ratio of 0:1, and 1:1 were stained with Oil Red O and visualized by microscopy, representative images from Oil Red O-stained cells are shown; scale bar, 100 μm. **(B)** The cells were harvested and total RNA was isolated for analysis of adipogenic, browning, and inflammatory markers by RT-qPCR. **(C-D)** For cells co-cultured at a 0:1 and 1:1 ratio, levels of **(C)** cytokines and **(D)** chemokines in CM were measured. Data are presented as mean values ± SEM; statistical analysis was performed using **(B)** one-way ANOVA then Dunnett’s *post hoc* test or **(C, D)** unpaired t-test (n = 3); *p < 0.05, **p < 0.01, ***p < 0.001, and ****p < 0.0001. HM, AT-derived macrophages from HFD-fed mice; D3T3-L1, differentiated 3T3-L1 adipocytes.

Since macrophage-adipocyte crosstalk is crucial for the progression of obesity (45), we differentiated 3T3-L1 pre-adipocytes to adipocytes using our standard protocol and co-cultured HFD eAT-resident macrophages from HFD-fed mice for 72 h with the differentiated 3T3-L1 adipocytes at macrophage:3T3-L1 ratios of 0:1, 0.5:1, and 1:1. HM and 3T3-L1 are considered as inducer and target cells respectively in this set of co-culture. The CM was collected for subsequent assay of cytokine-chemokine levels and the cells were stained with Oil Red O, imaged with a bright field microscope, and harvested for analysis by RT-qPCR. There were intense stained and more compact red dots in 1:1 ratio of HM:D3T3-L1 culture compared to control 3T3-L1 adipocytes culture (**Figure 5A**). Thus the results of the Oil Red O staining indicated that when 3T3-L1 adipocytes co-cultured at a 1:1 ratio with macrophages from HFD-fed mice, it exhibited accumulation of more lipid droplets than control adipocytes (0:1 ratio) cultured in the absence of macrophages (**Figure 5A**). In the presence of macrophages from HFD-fed mice at ratios of 0.5:1 and 1:1, 3T3-L1 adipocytes showed upregulated expression of PPAR-γ, CEBPα, FASN, GLUT4, leptin, and TNF-α by RT-qPCR relative to adipocytes that were cultured alone, while the adipocyte browning marker PGC1α was downregulated at a 0.5:1 ratio (**Figure 5B**). Correspondingly, CM from the 1:1 co-culture contained significantly higher levels of cytokines IL-1β, IL-10, IL-12p70, and TNF-α and chemokines CXCL1, CXCL2, and CCL3 than did the control (**Figures 5C, D**). In this context, our outcome is similar to the study reported that adipogenesis takes place in adipogenic-angiogenic cell cluster at the early stage of obesity (46). Taken together, these results suggest that macrophages from HFD-fed mice induced synthesis and accumulation of lipids in adipocytes, adipogenesis, and inflammation and inhibited the expression of an adipocyte browning marker.

### T cells from HFD-fed mice modulated lipid accumulation, adipogenesis, and inflammation in 3T3-L1 adipocytes

**Figure 6:**
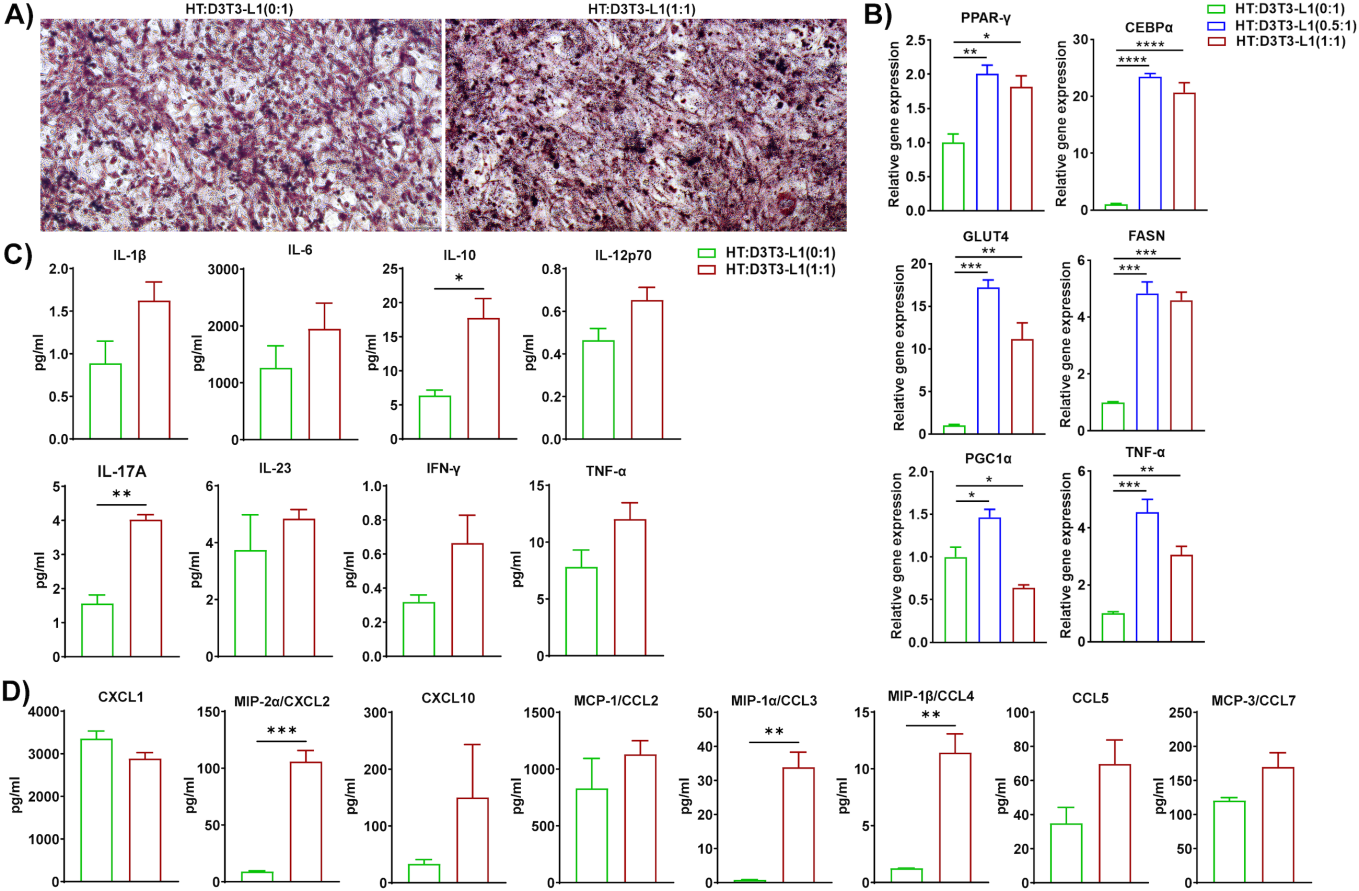
T cells from HFD-fed mice triggered lipid accumulation, adipogenesis, and inflammation in 3T3-L1 adipocytes. Mice were fed HFD for twelve weeks. T cells were isolated from the SVF epididymal AT of HFD-fed mice and cultured 3T3-L1 pre-adipocytes were differentiated into adipocytes and the two cell types were co-cultured for 72 h at a ratio of 0:1, 0.5:1, and 1:1, with the 0:1 ratio serving as a control. CM was collected and stored for later determination of cytokine and chemokine levels and the cells were stained with Oil Red O and visualized by microscopy. Total RNA was isolated for analysis by RT-qPCR. **(A)** Representative images from Oil red O-stained 3T3-L1 adipocyte (0:1, and 1:1) (scale bar 100 μm). **(B)** RT-PCR analysis of adipogenic, browning, and inflammatory markers. **(C-D)** For cells co-cultured at a 0:1 and 1:1 ratio, levels of **(C)** cytokines and **(D)** chemokines in CM were measured. Data are presented as mean values ± SEM; statistical analysis was performed using **(B)** one-way ANOVA then Dunnett’s *post hoc* test or **(C-D)** unpaired t-test (n = 3), *p < 0.05, **p < 0.01, ***p < 0.001, and ****p < 0.0001. HT, AT-derived T-cells from HFD-fed mice; D3T3-L1, differentiated 3T3-L1 adipocytes.

IL-17A is a well-known inflammatory cytokine in the context of obesity (47). Studies reported that IL-17A inhibits adipogenesis via the inhibition of adipogenic transcription factors (48), and induces thermogenesis. We isolated T cells from the eAT of HFD-fed mice and differentiated cultured 3T3-L1 pre-adipocytes to adipocytes, then co-cultured the two for 72 h at a ratio of 0:1, 0.5:1, and 1:1. HT and 3T3-L1 are considered as inducer and target cells respectively in this combination of co-culture. We collected the CM from each culture to assay for secreted chemokines and cytokines, stained the cells with Oil Red O and visualized them by bright field microscopy, then harvested the cells and isolated total RNA for RT-qPCR analysis. Representative images from Oil red O staining showed that there were more dense and highly stained red dots in a 1:1 ratio of HT: D3T3-L1 culture compared to differentiated 3T3-L1 adipocytes culture. It indicated that adipocytes that were co-cultured 1:1 with T cells from HFD-fed mice contained more lipid than the 0:1 control (**Figure 6A**). When co-cultured at ratios of 0.5:1 and 1:1, T cells from HFD-fed mice and 3T3-L1 adipocytes exhibited elevated expression of PPAR-γ, CEBPα, FASN, GLUT4, and TNF-α relative to the 0:1 control by RT-qPCR, but expression of the adipocyte browning marker PGC1α varied, appearing to be upregulated at a co-culture ratio of 0.5:1 and downregulated at a ratio of 1:1 (**Figure 6B**). The CM of the 1:1 co-culture contained prominently higher levels of secreted IL-10, IL-17A, CXCL2, CCL3, and CCL4 than that of the 0:1 control (**Figures 6C, D**). In this context, we noticed that IL-17A, lipid accumulation, and thermogenic gene PGC-1α increased in the co-culture of HFD T cells and differentiated adipocytes. These outcomes are opposite as well as consistent with previous studies. Further, the beneficial roles of IL-17A in the context of obesity are balanced with the presence of other inflammatory cytokines (49). Overall, these data suggest that T cells from HFD-fed mice induced synthesis and accumulation of lipids in adipocytes, adipogenesis, and inflammation but inhibited the expression of browning markers in co-cultures containing higher ratios of T cells to adipocytes.

### HFD DCs fueled lipid synthesis, adipogenesis, and inflammation in 3T3-L1 adipocytes

**Figure 7:**
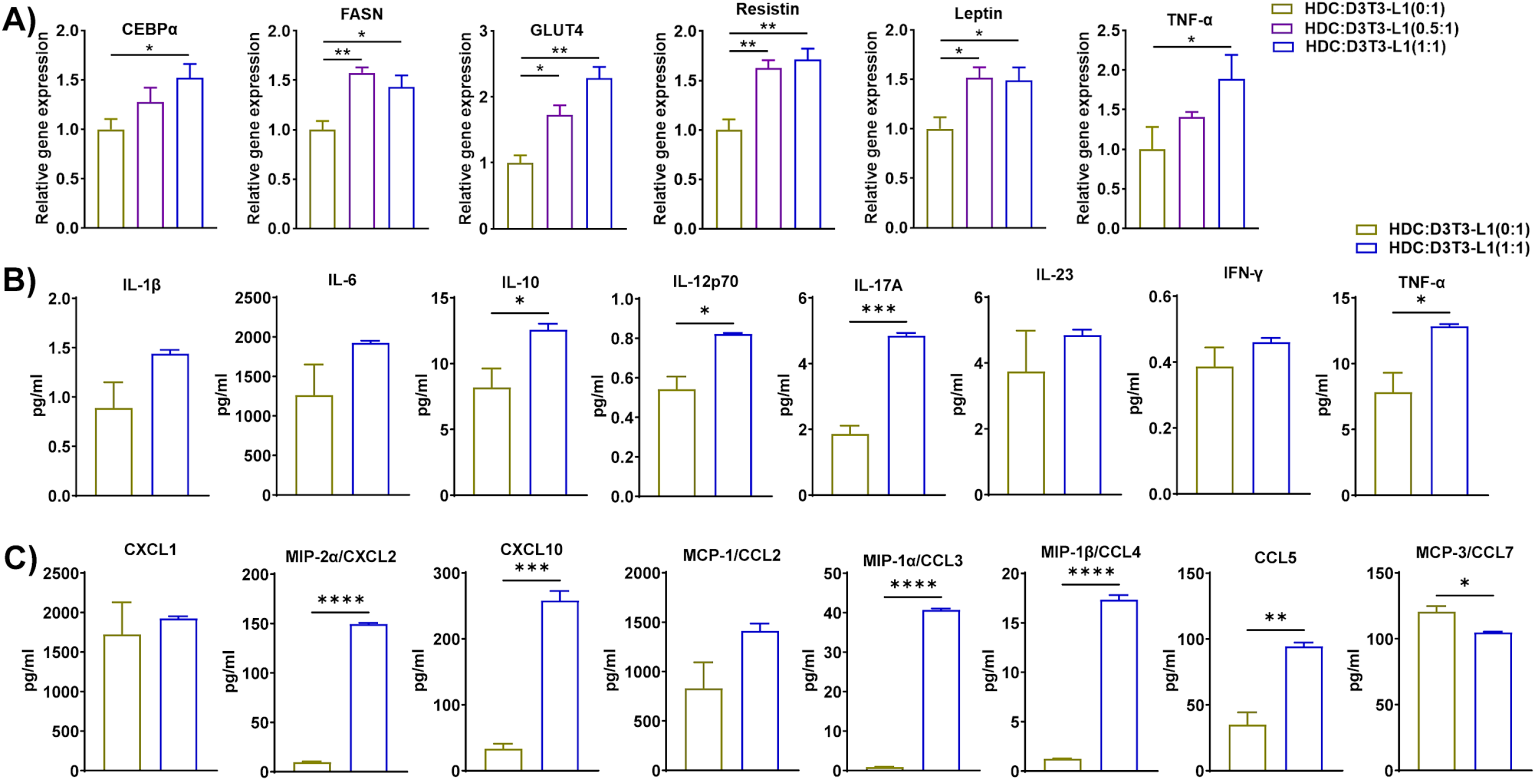
Dendritic cells (DCs) from HFD-fed mice accelerated lipid synthesis, adipogenesis, and inflammation in 3T3-L1 adipocytes. Mice were fed HFD for twelve weeks. DCs were isolated from the eAT SVF of HFD-fed mice, 3T3-L1 pre-adipocytes were cultured and differentiated into adipocytes, and the two cell types were co-cultured for 72 h at a ratio of 0:1, 0.5:1, and 1:1, with the 0:1 ratio serving as a control. CM was collected and stored for later determination of cytokine and chemokine levels; the cells were harvested and total cellular RNA was isolated for **(A)** RT-qPCR analysis of adipogenic, and inflammatory markers. **(B, C)** For cells co-cultured at a 0:1 and 1:1 ratio, levels of **(B)** cytokines and **(C)** chemokines in CM were measured. Data are presented as mean values ± SEM; statistical analysis was performed using **(A)** one-way ANOVA then Dunnett’s *post hoc* test or **(B, C)** unpaired t-test (n = 3), *p < 0.05, **p < 0.01, ***p < 0.001, and ****p < 0.0001. HDC, AT-derived dendritic cells from HFD-fed mice; D3T3-L1, differentiated 3T3-L1 adipocytes.

Dendritic cells are recruited to the AT during HFD-induced obesity and are critical regulators of inflammation and insulin resistance (50). Specific subsets of inflammatory DCs in obese mice induce differentiation of Th17 cells and their accumulation in AT (51). To determine whether DCs in HFD-fed mice modulate the functions of adipocytes, we isolated DCs from the eAT of HFD-fed mice, differentiated 3T3-L1 pre-adipocytes to mature adipocytes *in vitro*, and co-cultured them for 72 h at DC to adipocyte ratios of 0:1, 0.5:1, and 1:1. HDC and 3T3-L1 are considered as inducer and target cells respectively in this group of co-culture. We collected CM from each culture for analysis of chemokine and cytokine secretion by bioplex ELISA assay harvested the cells, isolated total RNA, and performed RT-qPCR analysis of adipocyte markers. The co-culture of DCs from HFD-fed mice and 3T3-L1 adipocytes at a ratio of 0.5:1 and 1:1 showed upregulated expression of FASN, GLUT4, resistin, CEBPα, and TNF-α and leptin relative to control (0:1) cultures (**Figure 7A**). By bioplex ELISA, we found that the secretion levels of IL-10, IL-12p70, IL-17A, and TNF-α in the CM of the 1:1 DC to adipocyte co-culture was prominently higher than that of the control (**Figure 7B**), while the levels of chemokines CXCL2, CXCL10, CCL3 CCL4, CCL5, and CCL7 increased (**Figure 7C**). Taken together, these results suggests that DCs from HFD-fed mice induced lipid synthesis, adipogenesis, and inflammation in adipocytes and stimulated infiltration of other immune cells.

### Macrophages, T cells, and DCs from HFD-fed mice induced differentiation of 3T3-L1 pre-adipocytes into adipocytes even in the absence of external differentiation agents

**Figure 8:**
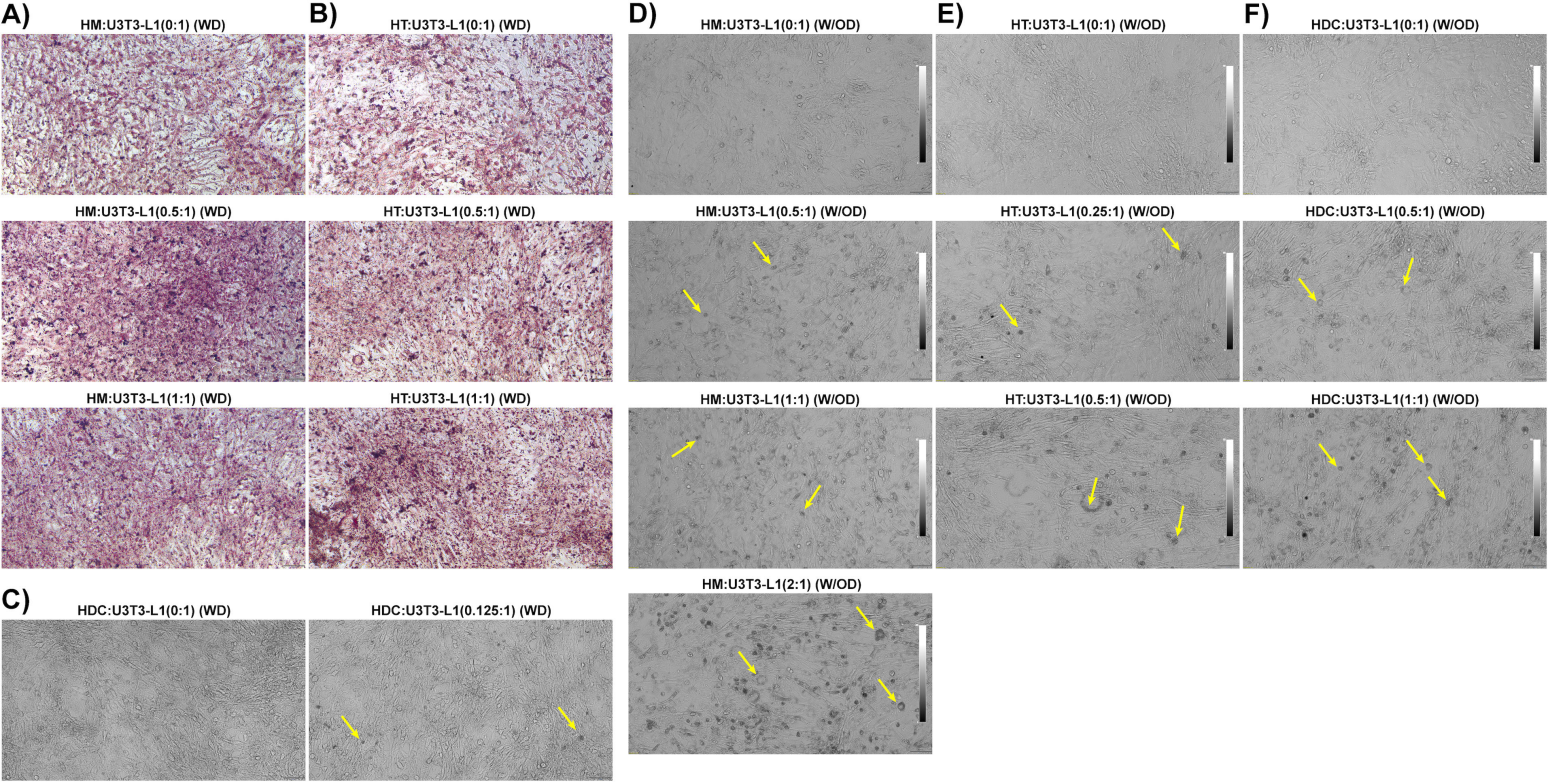
Macrophages, T cells, and dendritic cells from HFD-fed mice directly promoted differentiation of pre-adipocytes to adipocytes. Mice were fed HFD for twelve weeks. Macrophages, T cells, and dendritic cells (DCs) were isolated from the eAT SVF of HFD-fed mice and were co-cultured for 6 days with confluent 3T3-L1 pre-adipocytes either with differentiation induction cocktail or without external differentiating agents; a 0:1 ratio was used as a control. The cells were visualized under a phase contrast microscope in unstained live condition and stained with Oil Red O and imaged under a bright field microscope. **(A-C)** 3T3-L1 cells were co-cultured in the presence of a differentiation cocktail with a combination of **(A)** HFD macrophages:3T3-L1 (0:1, 0.5, and 1:1 ratios), **(B)** HFD T cells:3T3-L1 (0:1, 0.5, and 1:1 ratios), and **(C)** HFD DCs:3T3-L1 (0:1, and 0.125:1 ratios). **(D-F)** 3T3-L1 cells were co-cultured in the absence of a differentiation cocktail with **(D)** HFD macrophages:3T3-L1 (0:1, 0.5, 1:1, and 2:1), **(E)** HFD T cells:3T3-L1 (0:1, 0.25:1 and 0.5:1), and **(F)** HFD DCs:3T3-L1 (0:1, 0.5:1, and 1:1). Representative images are shown; scale bars, 100 μm. Lipid-containing 3T3-L1 adipocytes are indicated by yellow arrows in phase contrast images. HM, AT-derived macrophages from HFD-fed mice; HT, AT-derived T cells from HFD-fed mice; HDC, AT-derived dendritic cells from HFD-fed mice; U3T3-L1 (WD), 3T3-L1 pre-adipocytes with differentiation cocktail (IBMX, dexamethasone, and insulin); U3T3-L1 (W/OD), 3T3-L1 pre-adipocytes without differentiation cocktail.

Next, we tested whether HFD eAT-derived macrophages, T cells, and DCs were capable of providing a sufficient stimulus to induce the differentiation of pre-adipocytes to adipocytes *in vitro* without any external differentiation agents. First, we co-cultured eAT-derived macrophages, T cells and DCs from HFD-fed mice for six days with 3T3-L1 pre-adipocytes in the immune cell:3T3-L1 ratios of 0:1, 0.5:1, and 1:1 and 0:1, 0.125:1 in the presence of differentiation cocktail (isobutyl methylxanthine, dexamethasone, and insulin), following our differentiation protocol (WD). The HM, HT, and HDC are considered inducers, and U3T3-L1 (WD) cells are studied as target cells. We stained each culture with Oil Red O and used bright field microscopy and imaging to visualize the cells. We observed that the 0.5:1, and 1:1 ratios of macrophages to pre-adipocytes and T cells to pre-adipocytes exhibited more intense ORO staining (red dots) than their respective control (**Figures 8A, B**). Similarly, on co-culture of DCs with 3T3-L1 pre-adipocyte (0.125:1), we observed a greater number of lipid droplet-containing cells (yellow arrows) that were visible under the phase contrast microscope than seen in control (0:1) cells (**Figure 8C**).

Next, we co-cultured eAT macrophage, T cells, and DCs from HFD-fed mice for six days with confluent 3T3-L1 pre-adipocyte in the immune cell: 3T3-L1 ratios of 0:1, 0.25:1, 0.5:1, 1:1, and 2:1 as per availability of the inducer cell population, this time in the absence of any exogenous differentiation induction cocktail (W/OD). The HM, HT, and HDC are considered inducers, and U3T3-L1 (W/OD) cells are experimented with as target cells. Interestingly, there were a negligible number of lipid droplet-filled cells in the control (0:1 ratio) cultures (**Figures 8D–F**). However, the number of lipid-containing cells increased proportionately with the increased ratio of macrophages, T cells, and DCs from HFD-fed mice (**Figures 8D–F**). Of all the combinations tested, the co-culture of macrophages:3T3-L1 cells at a 2:1 ratio showed the most dramatic increase in the number of lipid-containing adipocytes detected (**Figure 8D**, bottom image). We also analyzed genes related to inflammation and adipocyte differentiation using RT-qPCR from these last sets of co-culture of U3T3-L1 (W/OD) with HFD immune cells (**Supplementary Figure 1A–C**). Gene expression data also clearly depicted that inflammatory gene and adipocyte-specific gene expression amplified parallel with the increased ratio of HFD macrophage, T cells, and DC. These results strongly suggest that HFD AT-resident immune cells are themselves potent inducers of pre-adipocyte to adipocyte differentiation.

## Discussion

Obese adipose tissue (AT) associated with low-grade chronic inflammation is one of the major healthcare challenges that create a huge socio-economic burden worldwide. The primary outcome of obesity is aberrant deposition of fat, predominantly in visceral fat depots, expansion of adipocytes, and infiltration of immune cells in AT. While obesity accelerates metabolic syndrome and several autoimmune diseases and immune crosstalk in the AT is crucial for maintaining the chronic inflammatory state, the underlying mechanisms remain unclear. Thus, deciphering the pathophysiology of obesity-associated inflammation will be critical for the design of new, more effective therapeutics to combat the ongoing pandemic phase of obesity. To address this issue, we have developed a co-culture assay that involves combinations of immune cells from mice in the HFD-fed diet-induced obesity with either ND-fed mice immune cells or adipocytes in contact mode. We used epididymal AT resident immune cells (macrophages, T cells, dendritic cells) from HFD-fed mice as inducer cells and the macrophage, T cells, and SVF from eAT of ND-fed mice and cultured 3T3-L1 pre-adipocytes and adipocytes as target cells. We collected a conditioned medium for evaluation of cytokine and chemokine levels, stained cells with Oil Red O to evaluate lipid accumulation in adipocytes, harvested cells, and isolated total RNA for evaluation of gene expression by RT-qPCR. In contrast, previous studies utilized *in vitro* culture of bone marrow-derived macrophage, peritoneal macrophage, macrophage cell lines, and splenic T cells to mimic *in vivo* obese AT microenvironment. However, we employed AT resident immune cells *viz.*, macrophage, T cells and dendritic cells, SVF from obese and lean mice, 3T3-L1 pre-adipocyte and adipocyte to replicate closer to *in vivo* AT in the context of obesity. In this perspective, our study is novel and truly mimics an *in vivo* system. Further, DCs are less explored cell immune cell types compared to macrophage and T cells in the framework of obesity. In this context, the present study is also unique.

In our diet-induced obesity model, HFD comprised 1.36 times more energy per gram compared to ND. It has been shown that fat in HFD is a highly energy-dense substance which results in lesser post-prandial energy expenses compared to carbohydrates and protein (52). Moreover, the elevated carbohydrate and protein intake proportionately activate their oxidation rates. Unlikely, increment of fat intake does not influence fat oxidation and results in positive fat balance and storage in adipose depots (53). Additionally, dietary fat is accumulated very effectively (about 96%) as body fat mass (54), and 54.35% of 60% kcal from the fat of our chosen HFD is derived from lard. Further, lard contains 40% saturated fat (55). Intriguingly, a saturated fatty acid-rich diet accelerates obesity-related inflammation, and other metabolic syndrome including insulin resistance (56, 57). Previous studies also reported a positive correlation between the percentage of energy of fat content in diet and obesity in mice models (58). Our result showed that HFD mice consume lower food amounts (g) compared to ND nonetheless increasing adiposity. As per energy calculation, the HFD group truly consumes more energy from the diet, and the vital energy source is fat.

Immunometabolism is a promising area of research to combat various diseases. In the cellular aspect, adipose tissue macrophage phenotype shifted from anti-inflammatory to pro-inflammatory type during high-fat diet-induced obesity (59). Generally, free fatty acids in circulation are elevated in high-fat diet-induced obesity. Further, the role of different fatty acids in modulating macrophage phenotype and function has been reviewed (60). Intriguingly, plasma membrane phospholipid saturated/unsaturated fatty acid ratio is a critical regulator of macrophage phagocytic activity (61). Macrophages are robustly able to recognize, uptake, store, distribute, and utilize lipid molecules. In this context, scavenging receptor expression, lipid uptake, and inflammatory pathway activation are positively connected (62). Further, saturated fatty acid (SFA) in engulfed fatty acid exacerbated the pro-inflammatory genes and M1 macrophage polarization (63). Conversely, unsaturated fatty acid-induced anti-inflammatory genes and oxidative phosphorylation in M2 macrophage through AMP-activated protein kinase (AMPK) signaling pathway. Indeed, SFA is the ligand for toll-like receptors (TLRs) which subsequently stimulate inflammatory pathways (64). SFA-like palmitic acid (PA) elevated MCP-1, CXCL10, and IL-8 expression in macrophages and PA also increased the synthesis of IL-1β and TNF-α in lipopolysaccharide-treated macrophages (65, 66). In this context, inflammatory macrophages secrete cytokines viz. TNF-α, IL-6, IL-1β, IFN-γ, and IL-23, etc. and chemokines viz. CXCL10, MCP-1, etc., are very crucial for insulin resistance, activation of JNK, ERK1/2, NF-κB pathways, and recruitment of more inflammatory cells in AT (37). Our data suggest that HFD eAT-derived macrophages promoted the expression of pro-inflammatory chemokines and cytokines, fueled adipogenesis, and induced the expression of inflammatory markers in the eAT of ND-fed mice and cultured 3T3-L1 adipocytes. Furthermore, our data suggest that T cells and DCs isolated from the eAT of HFD-fed mice also induced lipid accumulation and inflammatory cytokines in 3T3-L1 adipocytes. Taken together, an image of obesity arises in which macrophages modulate T cells in a coordinated fashion to stimulate adipocyte differentiation and adipogenesis to sustain chronic inflammation. Thus, targeting AT macrophages may be an excellent way to combat obesity.

The accumulation and impaired egress of macrophages is known to play a leading role in AT inflammation during obesity (67, 68). Our data suggest that macrophages from HFD-fed mice induce the expression of transcription factors and secretion of inflammatory cytokines including TNF-α, IL-6, IL-1β, IFN-γ, IL-17A, STAT3, and NF-κB when co-cultured with T cells from ND-fed mice. GATA3 is a well-known transcription factor for T cells (69), the expression of which is positively associated with visceral obesity, obesity-related inflammation, and insulin resistance (70, 71). Interestingly, we observed that GATA3 was upregulated in the co-culture of macrophages from HFD-fed mice and T cells from ND-fed mice. These macrophages also stimulated the expression of CCL5, CCL7, CXCL10, CXCL2, and CXCL1 by T cells. These findings are corroborated by previous observations that pathophysiologically stimulated T cells secrete CCL3, CCR4, CCL5, and CXCL10 (72–74). The outcome of this experiment strongly suggests that macrophages from mice with HFD-induced obesity effectively modulate the T cells from non-obese mice towards an inflammatory state, thereby increasing inflammation levels in these mice.

Furthermore, the macrophages from HFD-fed mice induced the differentiation of pre-adipocytes from the SVF of ND-fed mice and elevated expression and secretion of levels of inflammatory cytokines and chemokines including IL-1β, IL-6, TNF-α, IFN-γ, leptin, IL-17A, CCL5, CCL4, and CCL7 into the culture medium. These results were validated by our observations that macrophages from HFD-fed mice elevated inflammation and adipogenesis when co-cultured with differentiated 3T3-L1 adipocytes. These macrophages also induced the adipocytes to increase storage of lipids, as detected by Oil Red O staining, consistent with increased expression of fatty acid synthase (FASN) in 3T3-L1 adipocytes. Macrophages from HFD-fed mice also induced 3T3-L1 adipocytes to increase expression of the genes of transcription factors involved in adipogenic differentiation, including PPAR-γ and CEBP-α, and common inflammatory genes IL-1β, TNF-α, and leptin, and reduced expression of adipocyte browning marker. While our data lend support to the notion that targeting macrophages is a therapeutic option for obesity, as suggested previously by other investigators (75), extensive further study will be needed to reach a prudent conclusion about the regulation of adipocyte browning by macrophages from mice with HFD-induced obesity. However, in the interim, we found that eAT-resident macrophages from HFD-fed mice can convert pre-adipocytes to adipocytes both in the presence and the absence of exogenous differentiation induction agents such as IBMX (3-isobutyl-1-methylxanthine), dexamethasone, and insulin. These results primarily support our hypothesis that macrophages from HFD-fed mice have the potential to induce differentiation of adipocytes.

Our results on HFD macrophages accelerate pre-adipocyte differentiation into mature adipocytes is not consistent with the previous study, which focused on macrophage culture supernatant TNF-α and IL-1β prohibited adipocyte differentiation (76). In our study, we used the contact mode of co-culture HFD macrophage, T cells, and DC with confluent undifferentiated 3T3-L1 cells for six days to determine the effects. It has been reported that factors like macrophage density, spatial restriction, and long duration of culture reduce inflammatory phenotype and induce alternative M2 macrophage phenotype (77, 78). It has been shown that an increased number of M2 macrophages induce adipocyte differentiation through hyperplasia (79). Spatial restriction might be a reason for different outcomes. In this context, a study demonstrated real-time adipogenesis that happens in the early phase of obesity progression inside the adipogenic-angiogenic cell clusters which are different from CLS (46). Thus, there is a chance of any alternative pathway activation which has not been explored till now. Thus, taken together, an in-depth, mechanistic study is required to reveal underlying pathways and prudent conclusions and might open the door for future study.

The second crucial cell type that infiltrates the AT during obesity is the T cell, whose migration to the AT is promoted in mice with HFD-induced obesity (25). The AT-infiltrated macrophage secretes more pro-inflammatory cytokines that invited several T cell sub-types. Infiltration of Th1, Th17, and CD8 T cells is remarkably enriched, but numbers of regulatory T cells are decreased in the AT of obese individuals (14). We isolated the total T cells from the eAT of HFD-fed mice and co-cultured them with macrophages from ND-fed mice or cultured 3T3-L1 adipocytes and pre-adipocytes. Relative to expression levels in macrophages from ND mice alone, their co-culture with AT T cells from HFD-fed mice induced expression of both M1 and M2 macrophage genes and dramatically increased expression of M1 macrophage inflammatory cytokines like TNF-α, IL-1β, and IL-6. These results suggest a potential role for eAT-derived T cells in inducing an inflammatory response in normal eAT macrophages during obesity. In this context, a previous study reported that CD8^+^ T cells in obese adipose tissue intensified macrophage activation and infiltration of the AT (80). Our findings convey a similar concept. Moreover, we validated this finding by co-culturing T cells from HFD-fed mice with 3T3-L1 adipocytes, which resulted in upregulated expression of TNF-α, IL-17A, CXCL2, CCL2, CCL3, and CCL4. Interestingly, CXCL2, CCL2, and CCL4 also function as macrophage inflammatory proteins (MIP) that induce macrophage migration. In this co-culture set, we also observed upregulation of adipocyte differentiation markers like PPAR-γ, CEBPα, and FASN, downregulation of browning marker PGC1α, and elevated accumulation of intracellular lipid. This is consistent with previous observations that differentiating adipocytes secrete MIPs (22). Overall, our data suggest that in HFD-induced obesity, T cells stimulate adipocyte differentiation, fat synthesis, and secretion of MIP, which in part attract more macrophages and neutrophils to the AT to induce and promote chronic inflammation. Moreover, our data also suggest that HFD eAT-resident T cells from HFD-fed mice can convert pre-adipocytes to adipocytes both with and without exogenously added differentiation-inducing agents and clearly define these T cells as inducers of adipocyte differentiation. In this context, it has been reported that activated T cells decrease pre-adipocyte to adipocyte differentiation in transwell co-culture and use external differentiation agents (25). However, our result did not corroborate this study in part due to the difference in experimental setup with respect to the co-culture system and utilization of external differentiation chemicals.

The role of the well-known antigen-presenting cells, dendritic cells (DCs), in obesity, has not been explored to the same extent as macrophages and T cells, although a recent study showed that DCs activate T cells in the AT (12). Our results demonstrated that on co-culture with adipocytes, DCs from mice with diet-induced obesity accelerated adipocyte differentiation, increased expression of inflammatory genes like TNF-α and leptin, and stimulated the adipocytes to secrete inflammatory cytokines like IL-17A, TNF-α and chemokines CCL5, CCL4, CCL3, MCP-3, CXCL10, and CXCL2. Hepatic DCs with high lipid content are known to become more immunogenic to activate T cells, produce more TNF-α, and induce adipogenesis (81). Our data showing that eAT-resident DCs from HFD-fed mice able to differentiate pre-adipocytes to adipocytes even in the absence of exogenous differentiation factors corroborates the findings that DCs help to activate adipocytes in the eAT and induce an inflammatory response during obesity.

In the present study, IL-17A is remarkably upregulated in a few co-culture combinations like HFD macrophages: ND T cells, HFD macrophages: ND SVF, HFD T cells: 3T3-L1 adipocytes, and HFD dendritic cells: 3T3-L1 adipocyte along with bonafide pro-inflammatory cytokines. IL-17A is a well-known pro-inflammatory cytokine for several autoimmune diseases (82), and gaining attention in the context of obesity (19). It has been shown that the IL-23/IL-17 pro-inflammatory axis is elevated in obese women (83). IL-17 is stimulated by IL-6 during obesity (84). Furthermore, macrophage and DCs produce IL-1β and IL-23 also induces IL-17 from various T cell sub-types and innate lymphoid cells (85). We also observed that IL-1β, IL-6, and IL-23 were elevated in our co-culture combinations. Thus, these findings corroborated the other previous studies that IL-17A is induced either by a single or synergistic effect of IL-1β, IL-6, and IL-23 during obesity.

GLUT4 is an insulin-dependent glucose transporter on adipocyte and muscle cells, helps in glucose uptake and GLUT4 is decreased during obesity resulting in insulin resistance (86). Further, GLUT4 expression enhanced during adipocyte differentiation (87). It has been shown that PPAR-γ and its ligands, and agonists induce GLUT4 expression (88–90). In the present study, we noticed that GLUT4 is upregulated in 3T3-L1 adipocyte co-culture with HFD-derived macrophage, T cells, and DCs, and PPAR-γ is also upregulated in those co-cultures. Therefore, data at our disposal suggest that overexpression of PPAR-γ might induce GLUT4 in these cultures. However, an depth study is required to address the GLUT4 expression in co-culture in the context of obesity.

## Conclusions

This study suggests that macrophages, T cells, and DCs in the eAT collectively maintain low-grade inflammation in adipose tissue during obesity. The macrophages are primarily involved in maintaining inflammation and adipogenesis, while the T cells induce adipogenesis, and inflammation through the stimulation of chemokines that attract more inflammatory cell infiltrates to the eAT. The DCs in eAT primarily stimulate chemokine secretion from adipocytes and at least partially influence adipogenic differentiation and function of T cells in eAT during obesity. Although the secreted cytokines, membrane-bound ligands, and receptors are the operating factors for any cellular and molecular action in the contact mode of co-culture, however, the limitation of this method of co-culture approach is that both types of cells might produce some common genes and proteins. To overcome this limitation, we have selected some genes specific for the target cells like for macrophage – CD11b, F4/80, CD206, arginase; T cells – GATA3; adipocyte – CEBPα, FASN, GLUT4, leptin, resistin, PGC1α to explore the target specific alteration. On the contrary, activated immune cells and mature adipocytes are the source of common cytokines like TNF-α, IL-6, and IL-1β. In this context, transwell membrane separated co-culture and culture with conditional media by blocking the targeted cytokines by antibody will be contributory.

## Data Availability

The raw data supporting the conclusions of this article will be made available by the authors, without undue reservation.

## Supplementary material

Supplementary Figure 1 Macrophages, T cells, and dendritic cells from HFD-fed mice altered the gene expression of pre-adipocytes related to inflammation and differentiation. Mice were fed HFD for twelve weeks. Macrophages, T cells, and dendritic cells (DCs) were isolated from the eAT SVF of HFD-fed mice and were co-cultured for 6 days with confluent 3T3-L1 pre-adipocytes without external differentiating agents; 0:1 ratio was used as a control. Total RNA was isolated for analysis by RT-qPCR. **(A)** Analysis of TNF-α, IL-6, MCP1, and leptin gene expression by RT-qPCR in HFD macrophages: U3T3-L1 (W/OD) (0:1, 0.5, and 1:1). **(B)** Analysis of TNF-α, IL-6, resistin, and leptin gene expression by RT-qPCR in HFD T cells: U3T3-L1 (W/OD) (0:1, 0.25, and 0.5). **(C)** Analysis of IL-6, leptin, CEBPα, resistin, and FASN gene expression by RT-qPCR in HFD DC cells: U3T3-L1 (W/OD) (0:1, 0.25, and 0.5). Data are presented as mean values ± SEM; statistical analysis was performed using one-way ANOVA then Dunnett’s *post hoc* test (n = 3), *p < 0.05, **p < 0.01, ***p < 0.001, ****p < 0.0001. HM, AT-derived macrophages from HFD-fed mice; HT, AT-derived T cells from HFD-fed mice; HDC, AT-derived dendritic cells from HFD-fed mice; U3T3-L1 (W/OD), 3T3-L1 pre-adipocytes without differentiation cocktail (IBMX, dexamethasone, and insulin).
