# Supplementary figures and images for: High-fat diet-induced adipose tissue-resident macrophages, T cells, and dendritic cells modulate chronic inflammation and adipogenesis during obesity
Source: Front Immunol. 2025 Jun 3;16:1524544. doi: 10.3389/fimmu.2025.1524544 (PMC12170627; doi:10.3389/fimmu.2025.1524544)

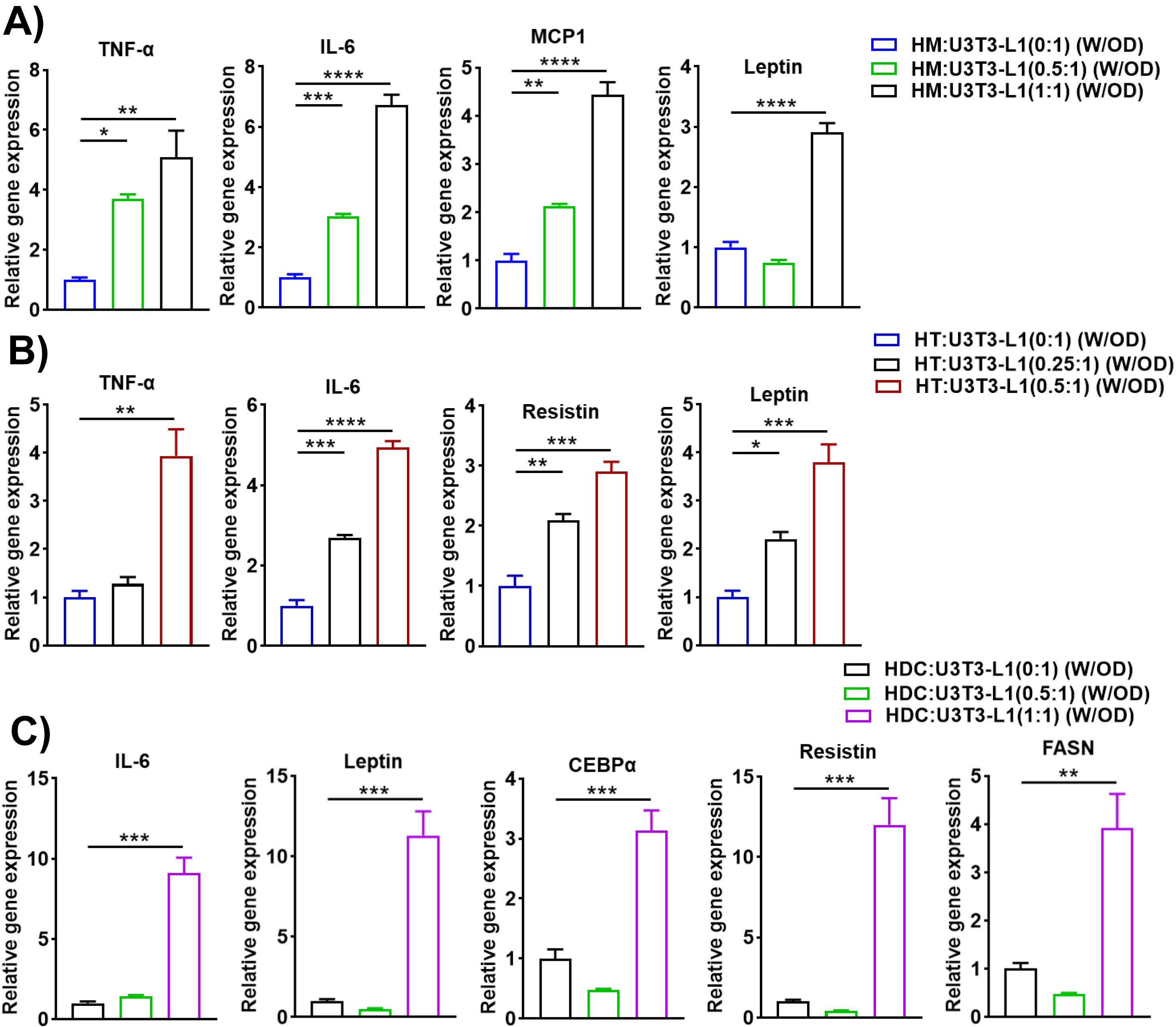

Supplement: Supplementary Figure 1 — Macrophages, T cells, and dendritic cells from HFD-fed mice altered the gene expression of pre-adipocytes related to inflammation and differentiation. Mice were fed HFD for twelve weeks. Macrophages, T cells, and dendritic cells (DCs) were isolated from the eAT SVF of HFD-fed mice and were co-cultured for 6 days with confluent 3T3-L1 pre-adipocytes without external differentiating agents; 0:1 ratio was used as a control. Total RNA was isolated for analysis by RT-qPCR. (A) Analysis of TNF-α, IL-6, MCP1, and leptin gene expression by RT-qPCR in HFD macrophages: U3T3-L1 (W/OD) (0:1, 0.5, and 1:1). (B) Analysis of TNF-α, IL-6, resistin, and leptin gene expression by RT-qPCR in HFD T cells: U3T3-L1 (W/OD) (0:1, 0.25, and 0.5). (C) Analysis of IL-6, leptin, CEBPα, resistin, and FASN gene expression by RT-qPCR in HFD DC cells: U3T3-L1 (W/OD) (0:1, 0.25, and 0.5). Data are presented as mean values ± SEM; statistical analysis was performed using one-way ANOVA then Dunnett’s post hoc test (n = 3), *p < 0.05, **p < 0.01, ***p < 0.001, ****p < 0.0001. HM, AT-derived macrophages from HFD-fed mice; HT, AT-derived T cells from HFD-fed mice; HDC, AT-derived dendritic cells from HFD-fed mice; U3T3-L1 (W/OD), 3T3-L1 pre-adipocytes without differentiation cocktail (IBMX, dexamethasone, and insulin). [file Image1.tif]
